# Piratical Medical Journals

**Published:** 1881-08-20

**Authors:** 


### *"PIRATICAL MEDICAL JOURNALS."*

The Editor of *Medical Bulletin*, in his August number, takes occasion to assault this journal, and two or three other respectable journals, for copying an article without giving credit therefor.

That it is wrong to take matter from another journal without due credit, we freely admit; and we further admit that every one who may be thus imposed upon may justly claim his rights and ask a correction of an error committed; but we do not admit that he is "morally obliged" to pre-judge the motives of the party who has offended, and to proclaim him a plagiarist and impostor, who, while "lacking brain for original work possesses cunning enough to appropriate that of others." Such is not the true and honorable way to correct an error. It is in itself antiscriptural and wrong, and may be done, as in the present case, in language so harsh and offensive as to constitute a wrong and a grievance even worse than that which he seeks to correct, and to place himself in a most unenviable attitude before his brethren of the press, who must either reply in the harsh and unbecoming manner which such an assault naturally provokes, or else treat the matter with silent contempt.

So far as this journal is concerned, we have never intentionally appropriated any matter from other journals without giving credit. Our printers are instructed to be careful in attaching the credit.

In the case of extracts they are expected to find the name of the journal at the bottom of the article and attach it. The case now complained of was of this kind, being only a part of an article the credit to which was unintentionally omitted by the printer. Such oversights, we doubt not, have occurred with perhaps all of our contemporaries, and we have occasionally had articles so copied from our journal: but we have never been so uncharitable as to believe that they were intentionally appropriated, or that any of our brethren of the press could be guilty of such conduct.

We have ever advocated a cordial and brotherly relation between the members of the medical press. We feel none of that envious or jealous spirit which would withhold a just credit to any one, and we have advocated at the editors' association, and in our journal, a spirit of brotherhood, and a co-operation in the advancing of every scheme and effort at reform in respect to journalism, to medical education and to medical progress. P.

